# Modeling of solvent-dependent conformational transitions in *Burkholderia cepacia *lipase

**DOI:** 10.1186/1472-6807-9-38

**Published:** 2009-05-28

**Authors:** Peter Trodler, Rolf D Schmid, Jürgen Pleiss

**Affiliations:** 1 Institute of Technical Biochemistry, University of Stuttgart, Allmandring 31, D-70569 Stuttgart, Germany

## Abstract

**Background:**

The characteristic of most lipases is the interfacial activation at a lipid interface or in non-polar solvents. Interfacial activation is linked to a large conformational change of a lid, from a closed to an open conformation which makes the active site accessible for substrates. While for many lipases crystal structures of the closed and open conformation have been determined, the pathway of the conformational transition and possible bottlenecks are unknown. Therefore, molecular dynamics simulations of a closed homology model and an open crystal structure of *Burkholderia cepacia *lipase in water and toluene were performed to investigate the influence of solvents on structure, dynamics, and the conformational transition of the lid.

**Results:**

The conformational transition of *B. cepacia *lipase was dependent on the solvent. In simulations of closed *B. cepacia *lipase in water no conformational transition was observed, while in three independent simulations of the closed lipase in toluene the lid gradually opened during the first 10–15 ns. The pathway of conformational transition was accessible and a barrier was identified, where a helix prevented the lid from opening to the completely open conformation. The open structure in toluene was stabilized by the formation of hydrogen bonds.

In simulations of open lipase in water, the lid closed slowly during 30 ns nearly reaching its position in the closed crystal structure, while a further lid opening compared to the crystal structure was observed in toluene. While the helical structure of the lid was intact during opening in toluene, it partially unfolded upon closing in water. The closing of the lid in water was also observed, when with eight intermediate structures between the closed and the open conformation as derived from the simulations in toluene were taken as starting structures. A hydrophobic β-hairpin was moving away from the lid in all simulations in water, which was not observed in simulations in toluene. The conformational transition of the lid was not correlated to the motions of the β-hairpin structure.

**Conclusion:**

Conformational transitions between the experimentally observed closed and open conformation of the lid were observed by multiple molecular dynamics simulations of *B. cepacia *lipase. Transitions in both directions occurred without applying restraints or external forces. The opening and closing were driven by the solvent and independent of a bound substrate molecule.

## Background

*Burkholderia cepacia *lipase (BCL) formerly known as *Pseudomonas cepacia *lipase has been shown to be a useful enzyme which catalyzes a broad range of different reactions in water and non-polar solvents under mild conditions. BCL is a highly selective catalyst for a broad range of substrates [1], including the kinetic resolution of racemic mixtures of secondary alcohols by hydrolysis in water or esterification in organic solvents [2-4]. All lipases have a similar architecture, the α/β hydrolase fold [5,6], which consists of parallel β-strands flanked by α-helices, and an active site with a catalytic triad consisting of Ser, His, and Asp/Glu [6,7]. The characteristic of most lipases is their activation upon binding to a hydrophobic interface [8]. Most lipases consist of a mobile element at the surface, a lid, which covers the active site [9]. The lid is opening at a hydrophobic interface, making the active site accessible for substrates and enhancing the activity of the lipase [10,11]. Because the exterior of the lid is hydrophilic and its interior is hydrophobic, the hydrophobic surface of lipases increases upon lid opening [12]. Therefore, in non-polar solvents the open conformation was expected to be thermodynamically favored [13].

The crystal structure of BCL has been determined without a bound inhibitor in an open conformation of the lid. The lid consists of a helix-loop-helix motif with helix α4 (residues 118–127) and helix α5 (residues 134–150) which are linked to helix α6 (residues 160–166) [14]. BCL contains an essential Ca^2+^-ion site [15] which stabilizes a β-hairpin (residues 214–228) [16]. While no experimental structure of BCL in the closed conformation is available, a homologous lipase from *Burkholderia glumae *(BGL) [PDB: 1QGE] [17] with a sequence identity of 84% has been crystallized in the closed conformation. The cores of both homologous lipases have an identical structure with a root-mean squared deviation (RMSD) of backbone atoms of 0.7 Å, while the main structural difference between the two conformations is the position of the lid (residues 118–150) with a RMSD of 5 Å. The length of helix α5 in BCL (residues 134–150) was identified to be longer compared to BGL (residues 136–150), and the length of helix α6 in BCL (residues 160–166) was shorter in BGL (residues 156–166). Thus, the X-ray structures of open BCL and closed BGL suggests that the interfacial activation of BCL involves a motion of helices α4 and α5 [14] and a partial unfolding of helix α6 in BGL. However, the mechanism of lid opening is still discussed. Several mechanisms of interfacial activation of lipases have been supposed. The substrate theory suggests that the lid opening is induced by binding of a substrate [18,19], while the enzyme theory suggests that the lid opens by adsorption to a hydrophobic interface [13]. Despite the fact that structures of the closed [9,13,17,20] and the open conformation [20-25] of several lipases are known, a complete description of the mechanism of lid opening and closing is still lacking. The identification of mobile elements was possible by the availability of open and closed structures, but attempts to obtain any intermediates between the closed and open conformation [25] and conformational transitions have not been successful in experiment. Therefore, short molecular dynamics simulations of different lipases have been reported to get more insight into the conformational transition of the lid [26-31]. However, the time-scale of conformational transition of the lid from the closed to the fully open conformation was not reached in short molecular dynamics simulations. Therefore, methods like essential dynamics [32], normal mode analysis [33], and Brownian dynamics simulations [34] were used to study the lid opening in non-polar solvents.

To investigate the solvent-dependent motions of the mobile lid (residues 118–150) and the β-hairpin (residues 214–228) of BCL, multiple molecular dynamics simulations of the open crystal structure and the closed homology model of BCL were performed in water and toluene without using restraints or external forces. Toluene was used as a typical non-polar solvent which is widely used in lipase-catalyzed conversions. It has been shown that BCL is stable in toluene without significant loss of activity. The details of the conformational pathway were examined, to distinguish whether the transition occurs via a rigid-body movement or via a partial or complete unfolding, and bottlenecks in the lid opening and closing were identified.

## Results

Three simulations of 15–30 ns with different starting velocity distributions were performed for four enzyme-solvent systems, the closed homology model and the open crystal structure of BCL in water and toluene. The observed conformational transitions of the lid were dependent on the solvent, while all three simulations of the same enzyme-solvent system showed essentially the same transition. In eleven simulations, the Ca^2+^-binding site was stable, while in one simulation of closed BCL in water the Ca^2+^-ion left the binding pocket after 26 ns of simulation time.

### Analysis of crystal structure and homology model

All available crystal structures of BCL have been obtained in their open conformation [14,23,24,35,36] showing only small root mean squared deviations (RMSD) of backbone atoms in the range of 0.2–0.5 Å. In all crystal structures there are crystal contacts between helix α4 (residues 118–127) and helix α9 (residues 243–256) of a second monomer, as well as between residues 217–218 and 223–225 of the β-hairpin and residues 130–138 of helix α5 of the lid of a second monomer (additional file 1). The crystal contacts are stabilized by electrostatic interactions between the negative potential of the lid and the positive potential of the β-hairpin (data not shown). In addition, hydrophobic contacts were observed between helix α5 and its equivalent of a second monomer at residues 142 to 149. In contrast, there are no crystal contacts between the lid and the β-hairpin in the crystal structure in the closed conformation of the homologous *Burkholderia glumae *lipase (BGL) [PDB: 1QGE] [17], which was used as template for the homology model of closed BCL. The sequences of BCL and BGL are highly similar with a sequence identity of 84%. In the lid, only residues 125, 128, 129, 148, and 150 are different.

**Figure 1:**
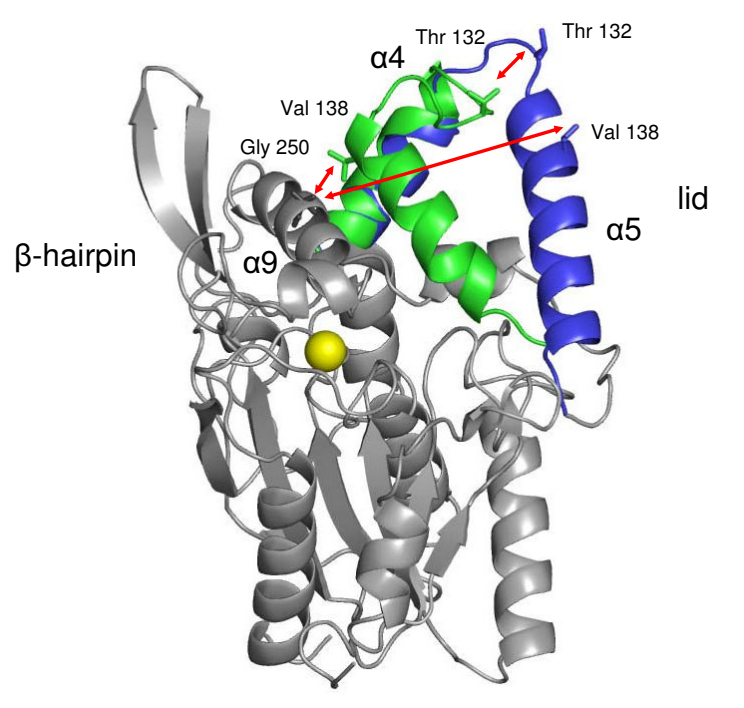
**Structure of open and closed BCL**. The open and closed conformation of BCL is shown in a cartoon representation. The lid of the open crystal structure of BCL [PDB: 3LIP] is colored blue and of the closed homology model green, the sphere of the Ca^2+^-ion is colored yellow. The catalytic triad consisting of Ser, His, and Asp is colored red. For analysis distances were measured between atom C_α _of residue 132 from the simulated to the initial structure, respectively.

The homology model of closed BCL showed no significant differences to the core of the open crystal structure, but was different in the conformation of the lid. The major movement was expected for helix α5 and the loop between helices α4 and α5, while helix α4 slightly changed its orientation. Helix α5 of BCL increased in length in the open conformation (residues 134–150) as compared to the closed conformation (residues 136–150), while helix α6 decreased in length in the open conformation (residues 160–166) as compared to the closed conformation (residue 156–166) of BCL (figure 1), as already been observed in the closed conformation of BGL. Furthermore, small differences can be observed in the position of the β-hairpin and in other loops (residues 17–27 and residues 50–59). The quality of the closed homology model of BCL was analyzed by ProSA showing a Z-score of -5.9 and a local model quality similar to the template BGL with a Z-score of -6.7. In contrast, the Z-scores of both closed structures were slightly better as compared to the open crystal structure of BCL showing a Z-score of -4.9 and a local model quality with high energy at helix α5 of the lid.

The conformation of the lid was characterized by two distances: the movement of the whole lid was described by the distance between atom C_α _of residue 138 in helix α5 to atom C_α _of residue 250 in helix α9, which was 6.1 Å in the closed homology model and 24.5 Å in the open crystal structure. The movement of the loop between helices α4 and α5 was described by the distance of atom C_α _of residue 132 from the closed to the open conformation, which was 11.1 Å between the homology model and the crystal structure.

### Simulation of closed BCL in water

In all three simulations of closed BCL in water no significant conformational change was observed. The backbone RMSD of the conformation after 30 ns to the initial structure was 1.3 Å of the core and 2.0 Å of the lid. The core was stable, deviations were mainly observed at surface loops, especially at the β-hairpin (residues 214–228) and the loop between helices α4 and α5 of the lid (residues 129–133). The lid was slightly closing as indicated by the distance C_α _138-C_α _250 which decreased from 6.4 Å to 6.1 Å and the movement of the loop between helices α4 and α5 of the lid by a distance of 5.7 Å between atom C_α _of residue 132 from the simulated to the initial structure. The position of helix α5 (residues 134–150) was unchanged, helix α4 (residues 118–127) showed a movement by 2.7 Å at its C-cap, near the loop between helices α4 and α5.

**Figure 2:**
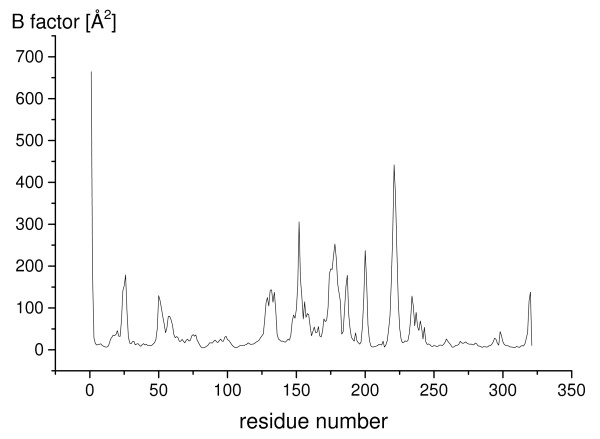
**Flexibility of closed BCL in water**. The flexibility of the closed conformation of BCL in water, indicated by calculated B-factors per residue [Å^2^], was calculated from the last 10 ns of simulation.

The calculated B-factors per residue identified helix α5, the loop between helix α4 and α5, the β-hairpin, helix α6 (residues 156–159), helix α7 (residues 170–178) and five further surface loops (loop 23–28, loop 48–61, loop 184–193, loop 198–203, loop 232–240) as the most flexible regions (figure 2). While the lid showed only a small movement, the flexible hydrophobic β-hairpin moved away from the lid by up to 9.7 Å (average of three simulations: 7.6 Å), measured by the distance of atom C_α _221 to its initial structure. Thus, the movement of the β-hairpin was independent of the movement of the lid.

### Simulation of open BCL in toluene

**Figure 3:**
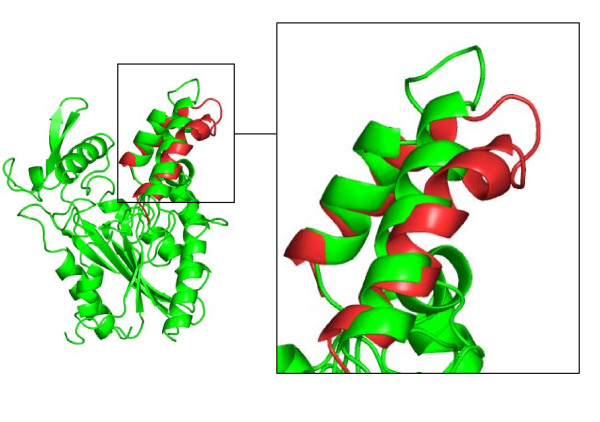
**Structure of open BCL in toluene**. The open crystal structure of BCL (green) in a cartoon representation showed a further lid opening after simulation of 30 ns in toluene (red).

**Figure 4:**
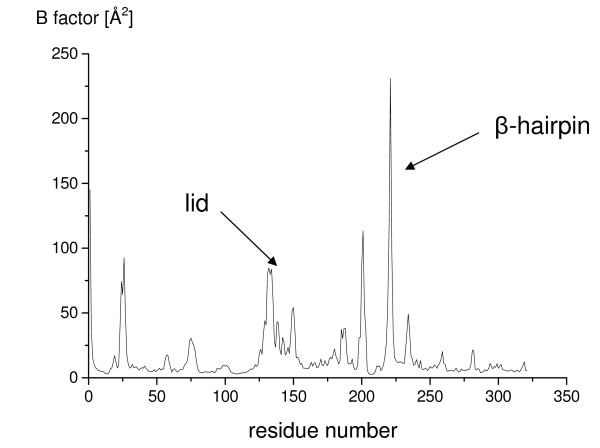
**Flexibility of open BCL in toluene**. The flexibility of the open conformation of BCL in toluene, indicated by calculated B-factors per residue [Å^2^], was calculated from the last 10 ns of the simulation in toluene.

In all three simulations of open BCL in toluene, the core was stable, while the lid opened slightly more (figure 3), as compared to the crystal structure. The backbone RMSD of the conformation after 30 ns to the initial structure was 1.0 Å of the core and 2.4 Å of the lid. The distance C_α _138-C_α _250 increased by 3 Å from 24.3 Å to 27.3 Å, indicating a further lid opening. The distance of atom C_α _of residue 132 in the loop between helix α4 and α5 from the initial structure to the simulated structure was 10.7 Å, indicating a major movement of the loop. In contrast to the simulations of the closed structure in water, the flexible β-hairpin did not shift. During the further lid opening, the hydrophobic surface increased by 80 Å^2 ^as compared to the crystal structure. The regions with highest flexibility as indicated by the calculated B-factors per residue were the lid, the β-hairpin, and further surface loops (residues 23–28, 185–189, 198–203, 232–240) (figure 4).

### Simulation of closed BCL in toluene

**Figure 5:**
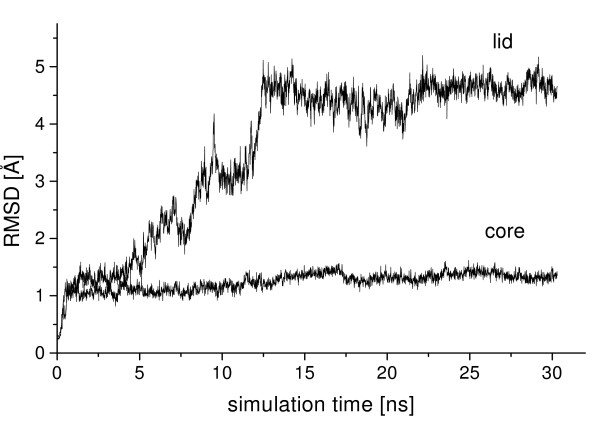
**RMSD in simulations of closed BCL in toluene**. Root mean squared deviations (RMSD) of backbone atoms of the core and the flexible lid as a function of time during simulation of 30 ns of closed BCL in toluene starting from the closed homology model.

**Figure 6:**
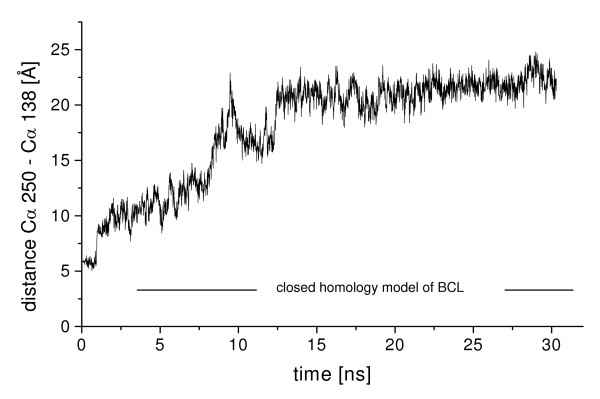
**Movement of the lid of closed BCL in toluene**. The movement of the lid of closed BCL as a function of time during the simulation of 30 ns in toluene was measured by the distance C_α _138-C_α _250. The lid was opening about 18 Å from the closed to the open conformation.

**Figure 7:**
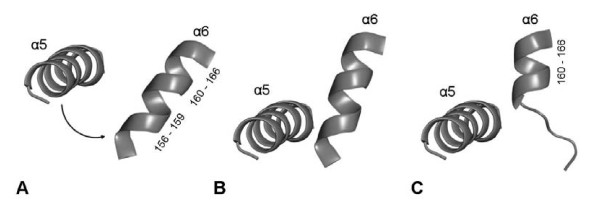
**Barrier during opening of the lid**. The movement of helix α5 (residues 134 to 150) was blocked by helix α6 (residues 156 to 166) during simulation of closed BCL in toluene (B) from their starting conformation (A). The unfolding of residues 156 to 159 of helix α6 would be necessary for the complete movement of the lid (C).

In all three simulations of closed BCL in toluene, the lid gradually opened [additional file 2], while the core of the protein was stable. The backbone RMSD of the conformation after 30 ns to the initial structure was 1.3 Å of the core and 4.8 Å of the lid. The RMSD to the initial structure continuously increased during the first 13 ns of simulation (figure 5) by a fast opening of the lid (figure 6), while the distance C_α _138-C_α _250 increased by 17 Å. Between 13 and 22 ns the lid slightly opened further by a distance C_α _138-C_α _250 of 2 Å, approaching its position in the nearly open conformation of the crystal structure until reaching a barrier (additional file 3). After gradually opening, the movement of the lid was blocked by helix α6 at a distance C_α _138-C_α _250 of 23.2 Å, near to its position in the crystal structure with a distance C_α _138-C_α _250 of 24.5 Å. The movement of helix α5 was blocked by the N-cap of helix α6 (residues 156 to 159) (figure 7) and it was not possible to overcome this barrier until the end of simulations. While in the simulation residues 156 to 159 were helical as in the homology model of the closed structure, helix α6 (residues 160–166) in the crystal structure of the open conformation was partially unfolded near its N-cap, leaving residues 156 to 159 in a loop structure (additional file 4), which would allow the further movement of helix α5.

**Figure 8:**
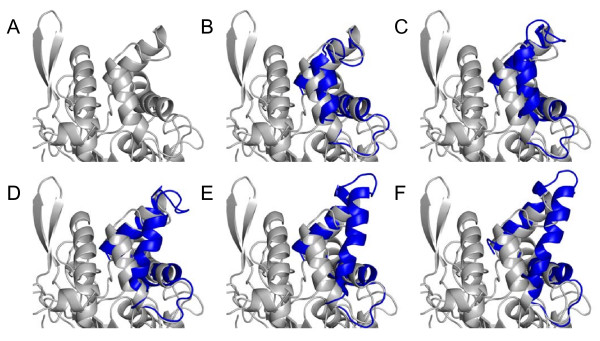
**Conformations of closed BCL during conformational transition**. Conformations of closed BCL (grey) in a cartoon representation during gradually lid opening (blue) in simulation of 30 ns in toluene (A) at the beginning and (B) after 3 ns, (C) 7 ns, (D) 10 ns, (E) 17 ns and, (F) 30 ns.

**Figure 9:**
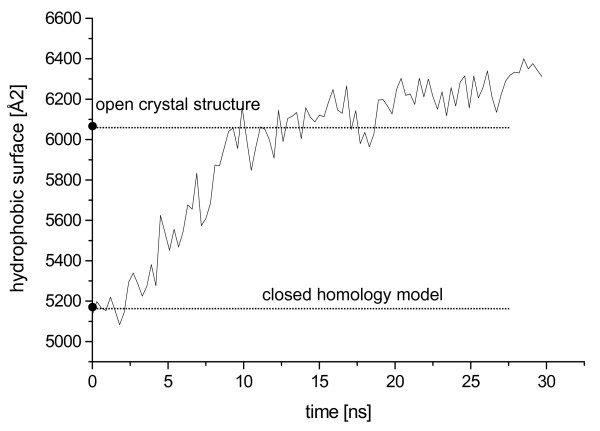
**Hydrophobic surface during conformational transition**. The hydrophobic surface of BCL during the 30 ns simulation of closed conformation in toluene is represented as a function of time. The hydrophobic surface increased by 1100 Å^2 ^by lid opening.

In all simulations in toluene, a salt bridge was formed between Asp121 at the N-cap of helix α4 and Arg258, which was not observed in the crystal structures of BCL and the homology model derived from BGL. This salt bridge prevented helix α4 from a movement in toluene. The opening of the lid was a hinge-type motion of a single helix α5 and the concomitant stretching of the flexible loop between helix α4 and α5. During the conformational transition the secondary structure of helix α5 was conserved. Thus, the lid opening involved conformational rearrangements of several secondary structure elements: a rigid body movement of helix α5 and a movement of the loop between helix α4 and α5, while helix α4 showed no significant movement (figure 8). The solvent accessible surface area (SASA) of the open crystal structure (13309 Å^2^) was 300 Å^2 ^larger than the SASA of the closed conformation of the homology model (12978 Å^2^). During lid opening, the SASA increased by 800 Å^2 ^to 13803 Å^2^. While the hydrophilic SASA decreased by 300 Å^2^, the hydrophobic SASA increased by 1100 Å^2 ^exposing a large hydrophobic patch of the lid to the non-polar solvent (residues Phe119, Val123, Val126, Val138, Ile139, Phe142, Val143, Val145, Phe146, and Leu149). The increase in hydrophobic SASA mainly occurred during the first 13 ns in correlation with lid opening (figure 9). While in the closed structure this hydrophobic patch was shielded from the solvent, making the protein surface more hydrophilic (additional file 5), in the open conformation the hydrophobic interior of the lid was exposed to the solvent.

The β-hairpin was very flexible, but showed no directed movement in contrast to the simulations in water.

### Simulation of open BCL in water

In the three simulations of open BCL in water, a partial lid closing was observed, while the core of the protein was stable. The backbone RMSD of the conformation after 30 ns to the initial structure of open BCL was 0.7 Å of the core and 3.1 Å of the lid. The distance C_α _138-C_α _250 decreased by 5.8 Å, from 24.4 Å to 18.6 Å. The distance of atom C_α _of residue 132 in the loop between helix α4 and α5 to its position in the open crystal structure increased to 12.3 Å. However, the fully closed conformation was not reached. Predominantly helix α5 was moving, while helices α4, α6, and α9 showed no movement. The movement of helix α5 was very slow and was not blocked by other structural elements. In contrast to the opening in toluene, helix α5 partially unfolded at its N-cap (residues 134–138) and changed from a straight to a more curved structure. As in the simulations of closed BCL in water, the β-hairpin showed a movement away from the lid by up to 7.2 Å (average of three simulations: 5.3 Å).

**Figure 10:**
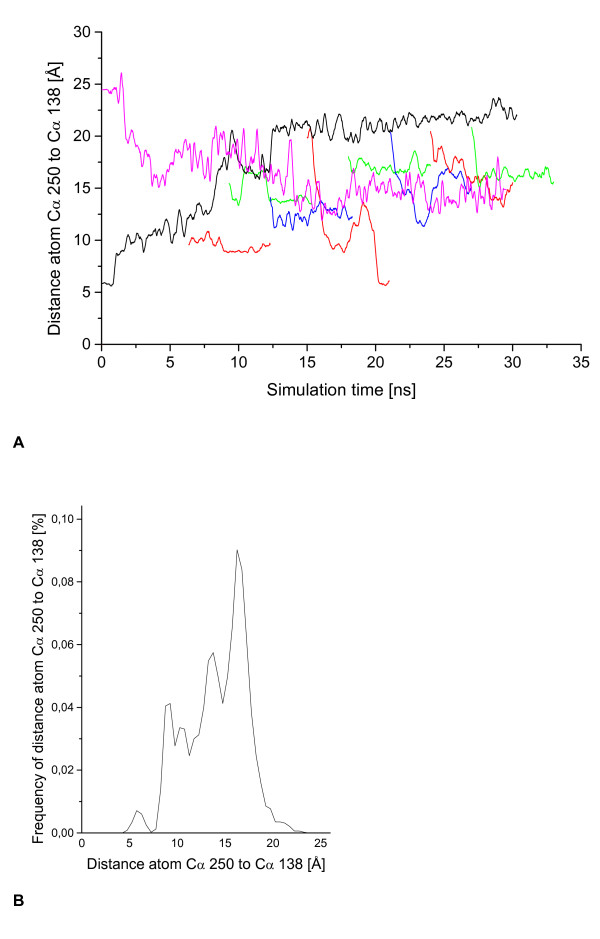
**Reverses simulations in toluene**. (A) After simulation of open BCL the lid opening of BCL in toluene was reversed in simulations of 6 ns in water, represented as a function of time. The open crystal structure (magenta) and several conformations from the previous lid opening (red, green, blue) in the simulation of closed BCL in toluene (black) were closing again after changing the solvent to water. The lid closing of BCL was measured by the distance between atoms C_α _138 and C_α _250. (B) The frequency of the distance between atoms C_α _138 and C_α _250 in the reversed movement of the lid in water was analyzed. Frequently occurring distances are 6, 10, 14, and 16.5 Å.

To investigate whether the solvent is the only driving force for the observed conformational transition, eight simulations in water were performed starting with intermediate structures taken from the simulation of the lid opening in toluene. Conformers were taken at 6, 9, 12, 15, 18, 21, 24, and 27 ns and simulated for 6 ns after changing the solvent from toluene to water (figure 10A). In all simulations, the lid was partially closing and the distance C_α _138-C_α _250 changed between 1.9 to 14.2 Å (table 1), indicating that the conformational transition can be reversed by changing the solvent. In one simulation, using the conformer after 15 ns as starting structure, the lid closed completely after 6 ns. There was no blocking of the lid by other structural elements in the simulations. It is quite remarkable, that for all simulations in water four preferred distances C_α _138-C_α _250 were identified at 6, 10, 14 and 16.5 Å (figure 10B).

**Table 1:** Reversed movement of the lid

**Conformation of BCL in toluene after [ns]**	**6.7**	**10**	**13.3**	**16.7**	**20**	**23.3**	**26.7**	**30**
**Distance C_α _138-C_α _250 before simulation [Å]**	12.5	17.9	17.7	20.3	19.2	21.9	21.8	22.3

**Distance C_α _138-C_α _250 after simulation [Å]**	8.9	13.6	12.5	6.4	17.0	14.8	15.2	15.4

**Δ distance C_α _138-C_α _250 [Å]**	3.6	4.3	5.2	13.9	2.2	7.1	6.6	8.8

The lid opening of BCL was reversed in simulations of 6 ns after changing the solvent from toluene to water, measured by distance C_α _138-C_α _250. The used starting conformations were selected from simulation of closed BCL in toluene after different times. The distance C_α _138-C_α _250 in the closed homology model was 6.1 Å.

### Coordination of Asp130

**Figure 11:**
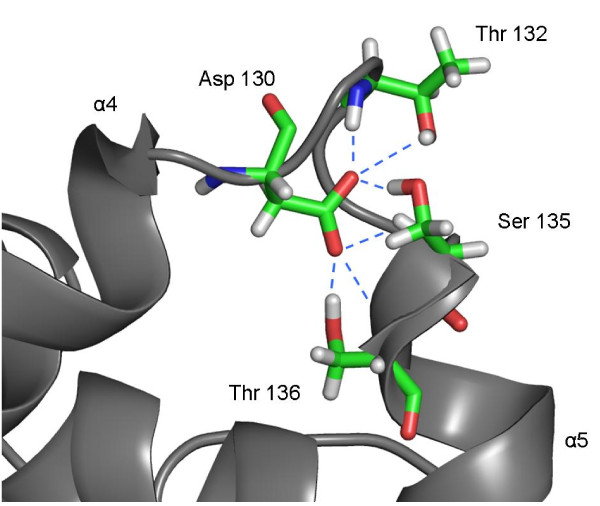
**Stabilization of Asp130 by hydrogen bonds**. After the simulation of BCL in toluene Asp130 in the lid was coordinated by six hydrogen bonds to backbone and side chains of Thr132, Ser135 and Thr136. The secondary structure of BCL is colored grey, hydrogen, carbon, nitrogen and oxygen atoms are colored white, green, blue and red, respectively.

In the crystal structures of BCL and BGL, the carboxylic group of the side chain of Asp130, located in the loop between helix α4 and α5, is coordinated by hydrogen bonds between the backbone and the side chain of Thr132, and the backbone of Ser135, stabilizing the conformation of the loop. During the simulations of open and closed BCL in water, the side chain of Asp130 was also coordinated by the backbone and the side chain of Thr132, and the backbone of Ser135, but these hydrogen bonds were partially lost during the simulation and replaced by hydrogen bonds to water molecules of the solvent. However, during simulations of closed BCL in toluene, Asp130 formed additional hydrogen bonds during lid opening and became fourfold coordinated by the backbones and side chains of Thr132 and Ser135. Furthermore, in the simulations of open BCL in toluene, Asp130 was sixfold coordinated forming two additional hydrogen bonds to backbone and side chain of Thr136 (figure 11). Thus, in toluene the loop between helix α4 and α5 was stabilized in the open conformation by the coordination of Asp130 in an extended hydrogen bonding to other amino acids of the loop.

## Discussion

The interfacial activation of lipases is associated with a conformational change of the lid [11,37]. The detailed analysis of the pathway of the conformational transition is not accessible in experiment. To get insight into the interfacial activation of BCL, multiple molecular dynamics simulations of the open and closed conformation of BCL in explicit solvent models of water and toluene were carried out without using restraints or external forces. An implicit organic solvent model was not used, because essential water molecules are supposed to be important to retain the flexibility of the lipase. Reproducibility of the observed properties was confirmed by performing three simulations for each system. The primary purpose was to apply molecular dynamics simulations examine the motion and conformational transition of the lid (residues 118–150) and the β-hairpin (residues 214–228) of BCL, which is not yet accessible to an experimental investigation.

For each system, a similar behaviour was observed: a fast opening of the lid in toluene starting with the closed structure, a closing of the open structure in water, and no major conformational changes in the simulation of the closed and open structure in water and toluene, respectively. Our major observations, the solvent-dependent opening and closing of the lid and the decoupling of the motions of lid and β-hairpin, were confirmed in an independent work in the group of P. Monsan (INSA, Toulouse) on simulation of conformational transitions of BCL: while lid opening occured at a water-hexane interface, closing of the lid was observed in water (I. André, personal communication).

### Simulation of closed BCL in water

In simulations of closed BCL in water, the lid stayed in its closed conformation. A slight further lid closing was observed which indicates that the homology model of closed BCL might not represent the closed conformation in solution. Furthermore, the lid showed a high conformational mobility in the absence of an oil-water interface which is supported by experimental observations. High B-factors of the lid were observed in all lipases in closed conformation [38-40], which led initially to the hypothesis that the lid of BGL can undergo a conformational transition. In crystallographic studies of closed *Thermomyces lanuginosa *lipase (TLL) in water, the lid was disordered [41], and in *Rhizopus delemar *lipase two different conformations of the closed lid were observed [42].

### Simulation of open BCL in toluene

In simulations of open BCL in toluene, the lid further opened. This was also observed in Brownian dynamics simulations of *Rhizomucor miehei *lipase (RML) [34]. At a lipid binding zone, which is comparable to a non-polar solvent, the lid further opened starting from the open crystal structure [43]. The further lid opening might be due to a different stabilization of the open structure in non-polar solvents as compared to the crystal structure. While in non-polar solvents, the lid is stabilized by an increase of hydrophobic surface, the conformation of the lid in the crystal is the result of energetic compromises to form stable contact interactions [44]. Upon molecular dynamics simulations of monomers in solution, starting with crystal structures, the structure is released from its crystal contacts and relaxed to a conformation close to that in solution [45]. The overall flexibility in simulations of open BCL in toluene was about half the flexibility of closed BCL in water, which is in agreement with a general observation of a decreased flexibility of proteins in non-polar solvents in experiment [46,47] and in molecular dynamics simulations [26,48-51].

### Simulation of closed BCL in toluene

In our simulations of closed BCL in toluene, the lid opening by 18 Å was observed by a rigid-body movement of helix α5 without unfolding of helices α4 and α5. A similar rigid-body hinge-type motion of a single helix was also concluded from the existence of a closed and open crystal structure of RML [21]. The lid opening of RML was observed in a low-dielectric medium, whereas in a high-dielectric medium no opening was observed [52]. In our simulations the hydrophobic surface of BCL increased upon lid opening, which is in agreement with a general observation of most lipases, where lid opening is associated with an increase in hydrophobic surface [12,20,52,53].

Previously, a partial opening of the lid has been investigated in simulations for different lipases. In molecular dynamics simulations in a continuum with different dielectric constants, lid opening was enhanced in a medium with a low dielectric constant, while the opening time of the lid increased for media with increasing dielectric constants [54]. In previous Brownian dynamics simulations, the lid of RML opened during 100 ns in a low-dielectric medium, while in a high-dielectric medium the lid stayed in its closed conformation for 900 ns [34]. In a 1 ns molecular dynamics simulation of RML in explicit water, the lid was highly flexible and a lid opening was concluded from essential dynamics analysis [27]. The partial lid opening was also observed for RML [43], *Humicola lanuginosa *lipase (HLL) [30], and *Candida rugosa *lipase (CRL) [55] in explicit solvents. In short molecular dynamics simulations of open BCL in water and vacuum, no conformational transition of the lid and a similar behaviour in different solvent environments was observed [28]. It was suggested that lid opening of HLL is a multi-step process involving more than two conformational transitions before a fully activated conformation is assumed [30]. This is in agreement with our simulations of BCL, where a barrier was identified, blocking the movement of helix α5 by the N-cap of helix α6. While the generated homology model of closed BCL and the template of closed BGL show a long helix α6 (residues 156 to 166), helix α6 is shorter in the open structure of BCL (residues 160 to 166), where Asp157 is replaced by Asn in the sequence of BCL. Thus, the unfolding of the N-cap of helix α6 (residues 156 to 159) would be necessary to make the complete movement of the lid towards the fully open structure feasible. However, this unfolding was not observed in the time scale in our simulations of 30 ns. The blocking of helix α5 by helix α6 seems to result from a different secondary structure of helix α6 in the open and closed structure.

A correlation of the lid movement with a surface loop was previously observed in restrained simulations of *Pseudomonas aeruginosa *lipase, which was suggested to trigger the lid opening [56]. In all our simulations of closed and open BCL in water, the β-hairpin drifted away from the lid, while it did not move in toluene, independent of the conformation of the lid. The conformational transition of the lid of BCL was not coupled to the β-hairpin or other surface loops, which was also not observed for other lipases [28,30,43,55].

Different mechanisms of interfacial activation have been already proposed. While in the 'substrate theory', changes in the conformation of the lid are dependent on a bound substrate molecule [18,19], in the 'enzyme theory' conformational changes on the enzyme are based on the adsorption to a lipid interface [13]. The lid opening in our simulations was only driven by the hydrophobicity of the solvent, where a hydrophobic solvent favored the opening of the lid, which supports the 'enzyme theory' and experimental observations. However, the solvent might not be the only reason leading to a complete opening of the lid, and we cannot exclude a possible influence of a substrate intermediate covalently bound to the catalytic serine. Several lipases have been crystallized in an open conformation in the absence of a bound inhibitor [57,14,36]. The lid opening of *Human pancreatic *lipase by water-miscible organic solvents without a bound substrate was also observed in experiment using antibodies [53], and a hydrophobic patch led to a conformational transition to the open conformation of CRL [58].

### Simulation of open BCL in water

The open conformation of BCL in water showed a partial lid closing during the simulations. However, lid closing in water was much slower than lid opening in toluene, and a complete movement of the lid to the closed conformation of the homology model was not observed. For CRL it was observed in experiment that the conversion between the open and closed conformation was very slow in aqueous solution in the absence of interfaces, which made the separation of the two conformations possible, while the exposure of CRL to a hydrophobic patch accelerated the transition [58]. However, a transition state between the closed and the open conformation could not be isolated. Short molecular dynamics simulations of the open conformation of BCL in water and vacuum demonstrated the high flexibility of the lid and the β-hairpin [28]. It was concluded from its high flexibility that the open structure of BCL becomes unstable in water leading to lid closing, however, a conformational transition from the open to the closed conformation was not observed.

The conformational transition of BCL from the closed to the open conformation of the lid in toluene could be reversed after changing the solvent from toluene to water. The analysis of eight simulations starting with intermediate positions of lid opening in toluene indicated that there are at least three local energy minima in addition to the closed crystal structure. In one simulation the lid closed completely, while in all other simulations a partially closing was observed. In contrast to the lid opening in non-polar solvents, the lid closing in water was a stepwise rather than a continuous process, associated with a partial unfolding and deformation of the helical structures in the lid.

### Coordination of Asp130

The solvent was the driving force of the opening and closing of the lid. Local unfolding by breaking hydrogen bonds was mediated by water molecules, while the formation of hydrogen bonds was driven by the non-polar solvent. The formation of a local hydrogen bond network was most prominent in the loop between helix α4 and helix α5, which seems to direct the conformational transition. Asp 130 plays a pivotal role in the hydrogen bond formation and stabilizes the open conformation of the lid in toluene. While Asp130 was sixfold coordinated by backbone and side chain atoms after simulation in toluene, which was closely related to the movement of the loop, these hydrogen bonds were lost in water by coordination of water molecules of the solvent, leading to a destabilization of the open structure. A pivotal role of single amino acids directing the lid opening might not be restricted to BCL, as observed in previous simulations of TLL, where a solvent-dependent switch of Arg84 in the lid was observed [25].

## Conclusion

The observed conformational transitions of the lid were dependent on the solvent. In simulations of closed BCL in water, no significant conformational change was observed, while in simulations of closed BCL in toluene the lid gradually opened. In simulations of open BCL in toluene, the lid opened slightly more than in the crystal structure, while in simulations of open BCL in water a partial closing of the lid was observed. In simulations of closed and open BCL in water, the hydrophobic β-hairpin moved away from the lid, while it showed no directed movement in simulations in toluene. Thus, the movement of the β-hairpin was independent of the movement of the lid.

## Methods

The crystal structure of open BCL was taken from the Protein Data Bank [PDB: 3LIP] [14] with a resolution of 2.0 Å by X-ray diffraction as initial structure for the simulations. There was no crystal structure of the closed conformation of BCL available. A homology model of closed BCL was built by the protein modeling server SWISS-MODEL [59] using the closed conformation of the homologous lipase from *Burkholderia glumae *(BGL) [PDB: 1QGE] [17] as template.

### Calculation of protonation states

pKa values and protonation states of titratable sites Arg, Lys, Asp, Glu and His at pH 7 were calculated using TITRA [60], based on the Tanford-Kirkwood model [61], using standard parameters. The solvent accessible surface area (SASA) of each residue was calculated by the program acc_run [62]. In toluene BCL was protonated assuming pH memory from the protonation states in water [49,63]. The electrostatic potential at pH 7 was calculated using DELPHI V. 4 [64] with a dielectric constant of the solvent of 80 and a molecule interior of 4. Atomic charges and radii were taken from the PARSE charge and radii files [65].

### System setup

Simulations were set up in XLEAP of the Amber 7.0 program package. Hydrogens were added as calculated by TITRA, His was protonated at H^δ^-position. Disulfide bridges were built from information in the crystal structure. The BCL structure, including 193 crystal water molecules, was solvated using the explicit TIP3 water model (dielectric constant 78.5) [66] and a non-polar solvent model of toluene (dielectric constant 2.4) [51] in a truncated octahedral box with a minimal distance of 14 Å between the box boundary and the protein. Three Na^+^-ions were added in XLEAP as counter ions to neutralize BCL for simulations.

### Molecular dynamics simulations

Multiple molecular dynamics simulations of the protein-solvent systems were performed using the AMBER 7 program package [67]. The all-atom AMBER force-field ff99 [68] was used to present the protein system and Ca^2+^-ions. The simulations were done in a truncated octahedral box under periodic boundary conditions. The Sander tool of AMBER 7 was used for minimization and simulations. Non-bonded interactions were calculated using the Particle-Mesh Ewald method to a cutoff distance of 10 Å and the SHAKE algorithm [69] was applied to constrain all bonds involving hydrogen atoms. The initial structures were energy minimized by applying 500 steps steepest descent and 50 steps conjugate gradient to relax clashes in the system. After the minimization steps the temperature was set to 300 K and the pressure to 1 bar under restrained conditions using a harmonic potential for all backbone atoms C, O and CA. The force constants were gradually decreased every 50 ps steps from 10, 5, 1 to 0.1 kcal/mol force constant, followed by a non-restrained simulation without additional external forces under periodic boundary conditions. Simulations were performed at 300 K using a time step of 1 fs. Temperature and pressure of the system were controlled using a weak coupling to an external heat bath [70] with a temperature coupling constant of 1.0 and a pressure coupling constant of 1.2. Three simulations, one up to 30 ns and two up to 15 ns, for the open and closed system in water and toluene were carried out using different starting atomic velocities from a Maxwell distribution to sample the conformational space. Each distribution was generated by a random number generator. The resulting trajectories were analyzed using PTRAJ of AMBER 7 after fitting the backbone atoms of the core (except residues 118–150 and residues 214–228) of each conformer to the initial structure. In PTRAJ the root-mean squared deviation (RMSD) of the backbone atoms between each conformer and the initial structure and between all conformers (2D-RMSD), atomic positional fluctuations relating to B-factors, and atom-atom distances were calculated.

### Structure analysis

Crystals were built up using SWISS-PDB Viewer and crystal contacts were analyzed by the WHAT IF web interface [71], including a 5.0 Å shell of symmetry related residues around the molecule, pairs of atoms were analyzed only between different asymmetric units. Secondary structure elements and the solvent accessible surface area (SASA) using a probe radius of 1.4 Å were calculated by DSSP [72]. Protein structures were visualized using PyMol 0.98 [73] and VMD [74]. Electrostatic potentials and hydrophobicity, using the hydrophobicity scale of Eisenberg [75], were mapped on the surface in PyMol 0.98. The quality of the homology model was analyzed using the ProSA-web interface [76].

## Authors' contributions

PT carried out the simulations, RDS contributed to the discussion of the results, JP was the principal investigator and directed the research. All authors read and approved the final manuscript.

## Supplementary Material

Additional file 1 **Crystal contacts of BCL**. In the crystal structure of BCL [PDB: 3LIP] [14] crystal contacts were observed. The β-hairpin (residues 214–228) (red) is in contact to the lid (residues 118–150) (yellow) of the next monomer. Click here for file

Additional file 2 **Interfacial activation of BCL**. Lid opening of closed BCL during interfacial activation in a 30 ns simulation of BCL in toluene. Click here for file

Additional file 3 **2D-RMSD in simulations of BCL**. The root mean squared deviation of every conformation to all other conformations as a function of time during simulation of closed BCL in 30 ns simulation in toluene is shown in the 2D-RMSD. A stable conformation after lid opening is indicated by the green area. Click here for file

Additional file 4 **Barrier during lid opening**. The lid opening of helix α5 was blocked by helix α6, indicated by the conformation at the end of the simulation of closed BCL in toluene (red), between the open conformation of the crystal structure (green) and the closed conformation of the homology model (blue). Click here for file

Additional file 5 **Hydrophobicity of BCL**. Hydrophobicity of (A) closed BCL and (B) open BCL mapped on the solvent accessible surface area calculated by DSSP, (hydrophobic parts red, hydrophilic parts white, active site yellow). Click here for file
