# Supplementary figures and images for: Modeling of solvent-dependent conformational transitions in Burkholderia cepacia lipase
Source: BMC Struct Biol. 2009 May 28;9:38. doi: 10.1186/1472-6807-9-38 (PMC2695465; doi:10.1186/1472-6807-9-38)

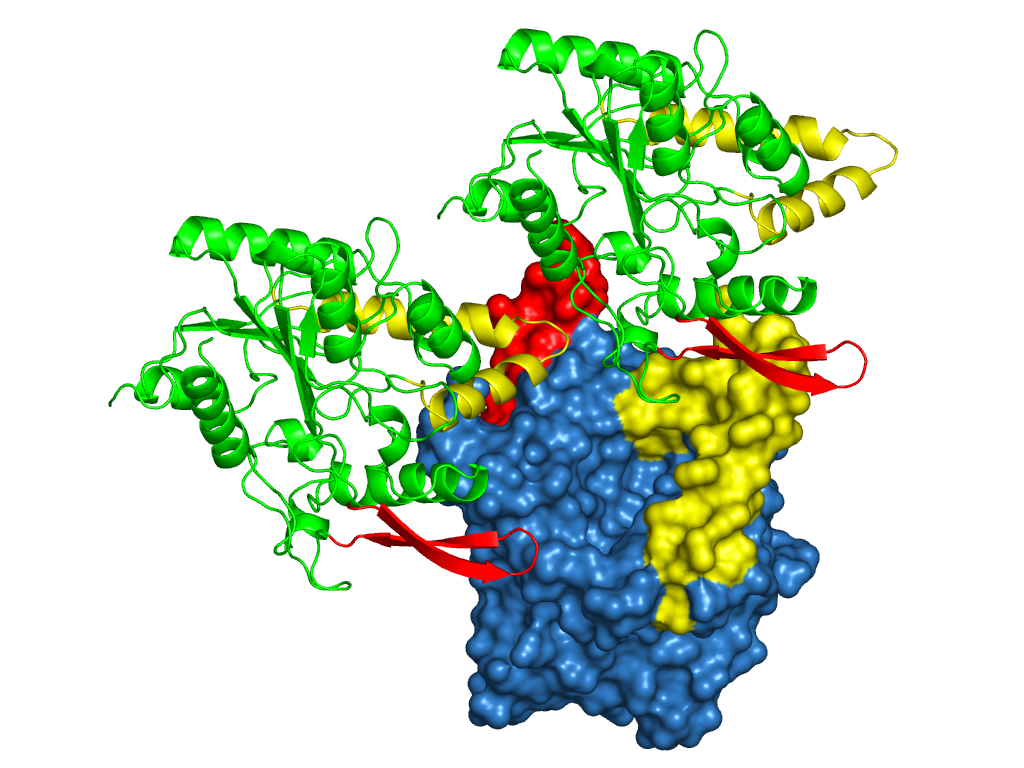


**Additional file 1**

Supplement: Additional file 1 — Crystal contacts of BCL. In the crystal structure of BCL [PDB: 3LIP] [14] crystal contacts were observed. The β-hairpin (residues 214–228) (red) is in contact to the lid (residues 118–150) (yellow) of the next monomer. [file 1472-6807-9-38-S1.doc]

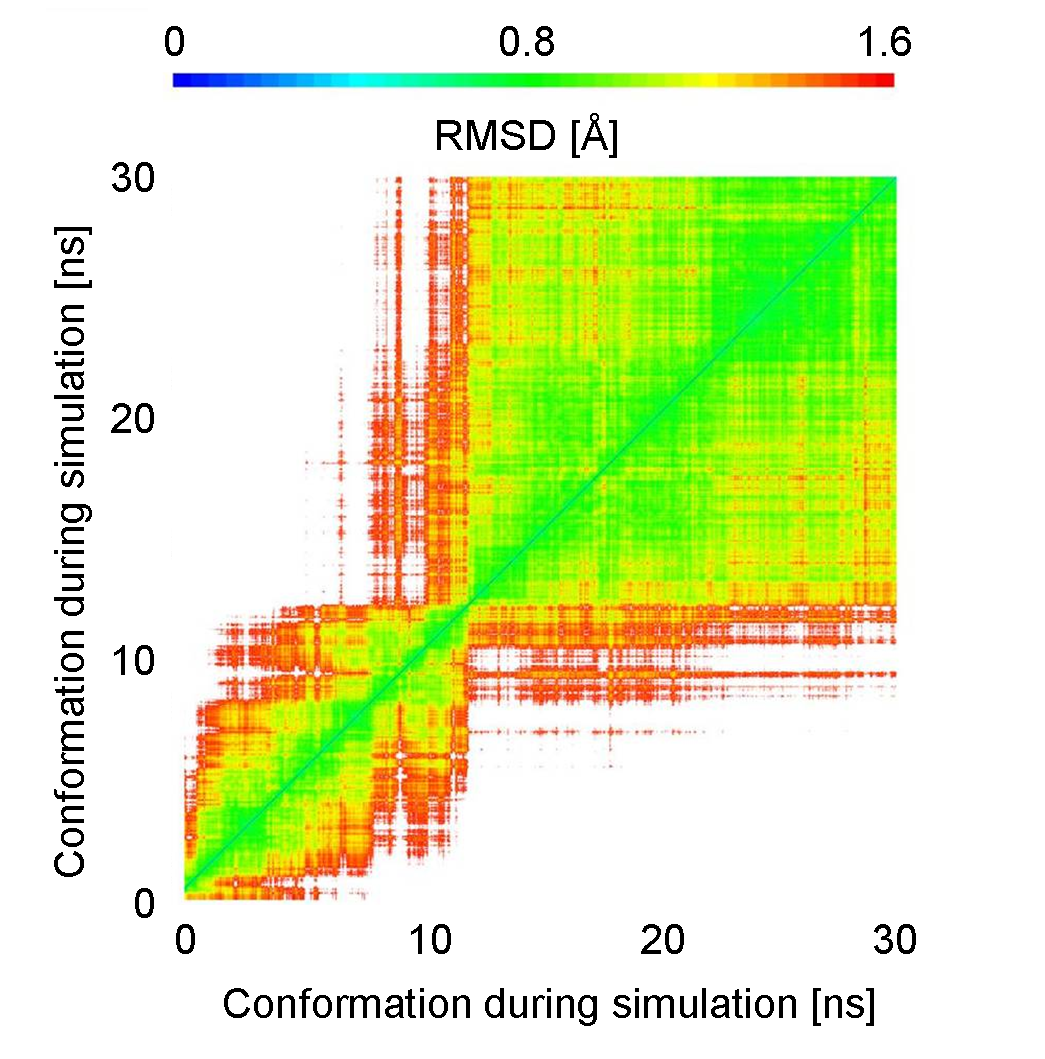


**Additional file 3**

Supplement: Additional file 3 — 2D-RMSD in simulations of BCL. The root mean squared deviation of every conformation to all other conformations as a function of time during simulation of closed BCL in 30 ns simulation in toluene is shown in the 2D-RMSD. A stable conformation after lid opening is indicated by the green area. [file 1472-6807-9-38-S3.doc]

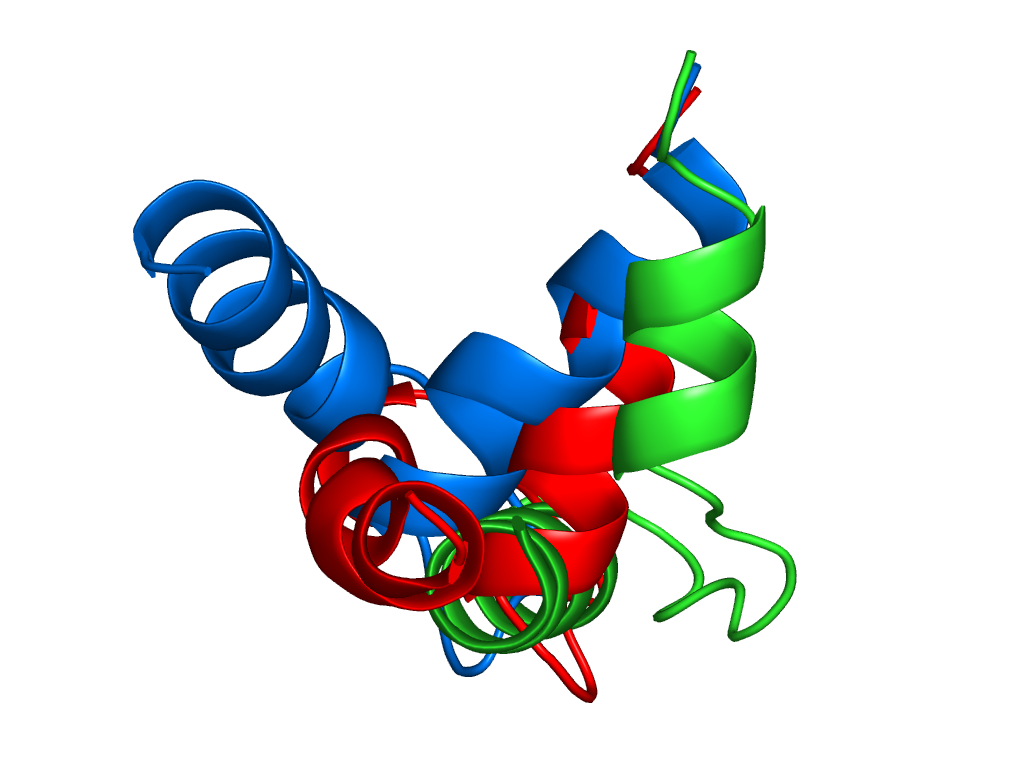

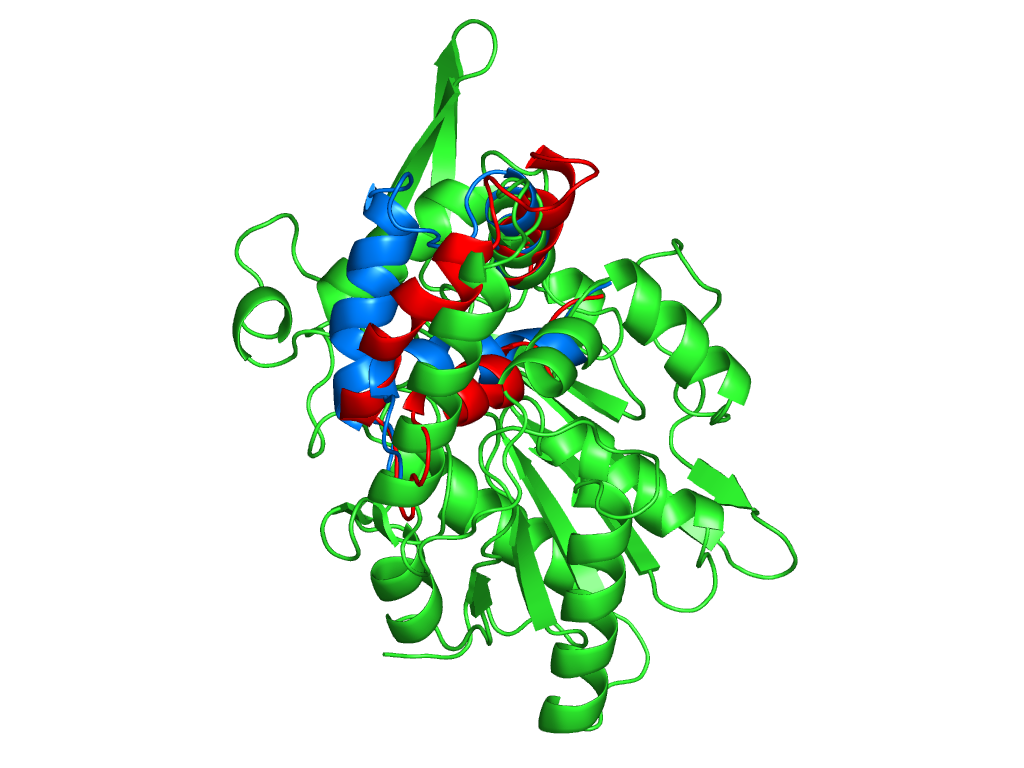


helix α5

helix α6

**Additional file 4**

Supplement: Additional file 4 — Barrier during lid opening. The lid opening of helix α5 was blocked by helix α6, indicated by the conformation at the end of the simulation of closed BCL in toluene (red), between the open conformation of the crystal structure (green) and the closed conformation of the homology model (blue). [file 1472-6807-9-38-S4.doc]

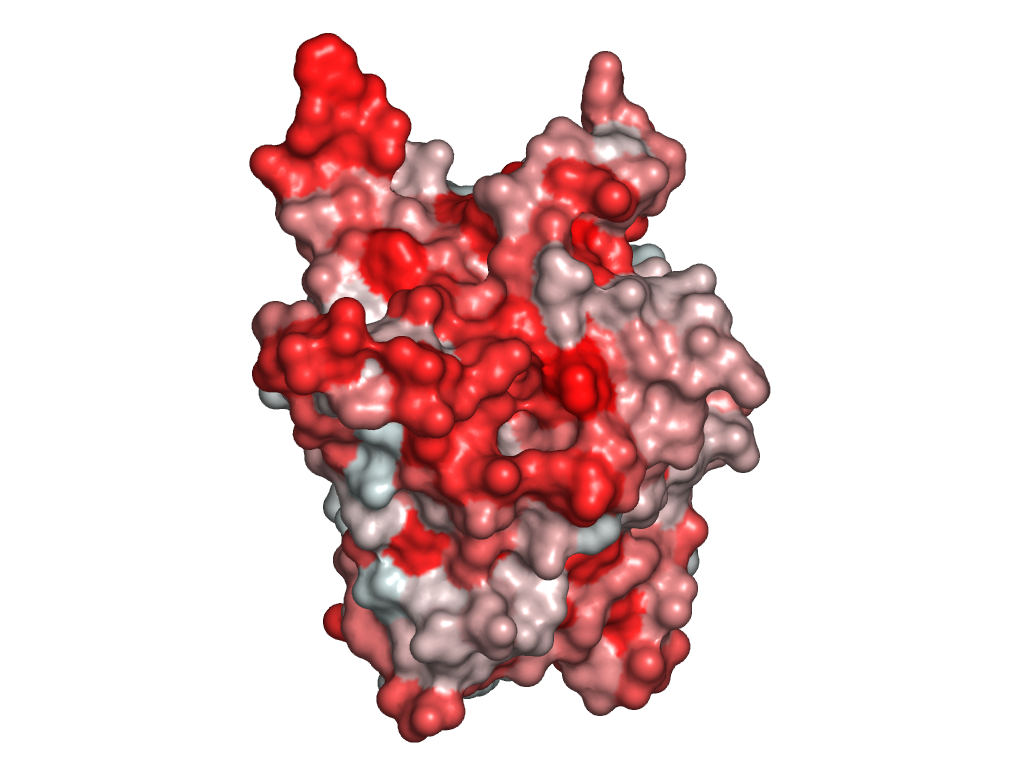

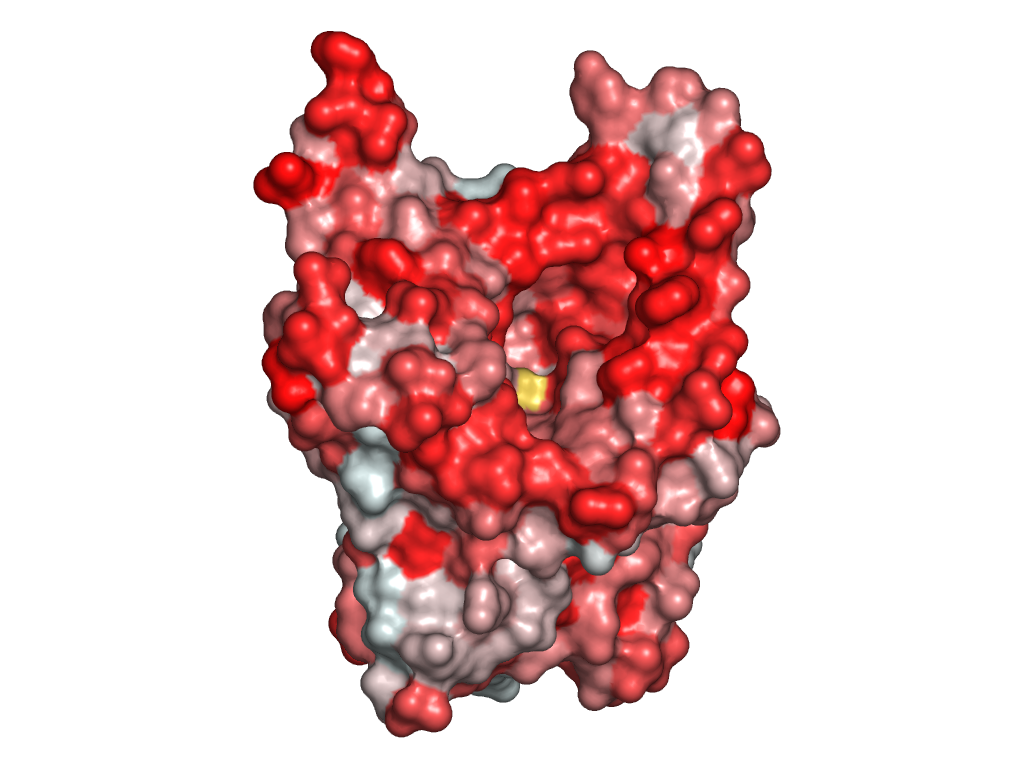


A

B

**Additional file 5**

Supplement: Additional file 5 — Hydrophobicity of BCL. Hydrophobicity of (A) closed BCL and (B) open BCL mapped on the solvent accessible surface area calculated by DSSP, (hydrophobic parts red, hydrophilic parts white, active site yellow). [file 1472-6807-9-38-S5.doc]
